# Co-evolution of transcription factors and their targets depends on mode of regulation

**DOI:** 10.1186/gb-2006-7-7-r62

**Published:** 2006-07-19

**Authors:** Ruth Hershberg, Hanah Margalit

**Affiliations:** 1 Department of Molecular Genetics and Biotechnology, Faculty of Medicine, The Hebrew University of Jerusalem, Jerusalem 91120, Israel

## Abstract

**Background:**

Differences in the transcription regulation network are at the root of much of the phenotypic variation observed among organisms. These differences may be achieved either by changing the repertoire of regulators and/or their targets, or by rewiring the network. Following these changes and studying their logic is crucial for understanding the evolution of regulatory networks.

**Results:**

We use the well characterized transcription regulatory network of *Escherichia coli *K12 and follow the evolutionary changes in the repertoire of regulators and their targets across a large number of fully sequenced γ-proteobacteria. By focusing on close relatives of *E. coli *K12, we study the dynamics of the evolution of transcription regulation across a relatively short evolutionary timescale. We show significant differences in the evolution of repressors and activators. Repressors are only lost from a genome once their targets have themselves been lost, or once the network has significantly rewired. In contrast, activators are often lost even when their targets remain in the genome. As a result, *E. coli *K12 repressors that regulate many targets are rarely absent from organisms that are closely related to *E. coli *K12, while activators with a similar number of targets are often absent in these organisms.

**Conclusion:**

We demonstrate that the mode of regulation exerted by transcription factors has a strong effect on their evolution. Repressors co-evolve tightly with their target genes. In contrast, activators can be lost independently of their targets. In fact, loss of an activator can lead to efficient shutdown of an unnecessary pathway.

## Abstract 2

Analysis of transcription regulatory networks in γ-proteobacteria reveals that repressors co-evolve tightly with their target genes, whereas activators can be lost independently of their targets.

## Background

**Figure 1:**
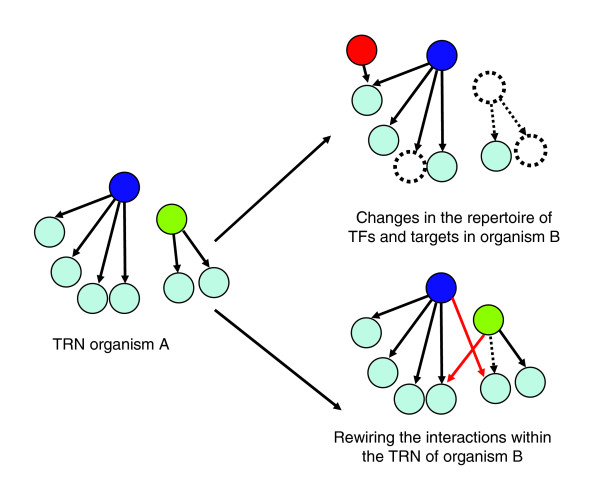
Schematic representation of the two parallel pathways by which the TRN evolves. Changes in the network may be achieved by removal or addition of TFs and/or targets, by rewiring of the network, or by both mechanisms.

The evolution of gene expression regulation plays an important role in the generation of phenotypic diversity. Organisms that share similar gene sequences may be phenotypically very divergent due to differences in regulation [1,2]. Gene expression is regulated at many different levels, among which the regulation of transcription initiation is prominent [3]. Initiation of transcription is regulated by transcription factors (TFs), which bind sequences within the promoters of their target genes and either activate or repress their transcription [4]. The combination of TFs and targets creates a complex network of regulatory interactions, termed the transcription regulation network (TRN). The nodes in this network are genes encoding TFs and target genes of TFs, and the edges are the regulatory interactions, pointing from TFs to their targets. The TRN evolves through two parallel processes [5-8]: the first process involves changing the regulatory interactions between TFs and targets, which can be described as rewiring of the network; and the second process involves the change in the repertoire of TFs and their targets, which can be described as the removal of nodes from the network and/or the addition of new nodes (Figure 1). In this paper we use the well characterized TRN of *Escherichia coli *K12 [9] as a reference, and compare all the genes within this network to the gene repertoires of many fully sequenced genomes of bacteria belonging to the same class as *E. coli *K12 (γ-proteobacteria). By focusing on bacteria that are relatively closely related to our reference organism we gain interesting insights regarding the dynamics of the evolution of transcription regulation, and demonstrate remarkable differences in the way in which the repertoires of activators and repressors evolve.

## Results and discussion

### Comparison of gene repertoires in TRNs of various organisms

To learn about the evolution of transcription regulation, we focused on the changes that occur in the gene repertoire of the TRN. We used the well characterized TRN of *E. coli *K12 [9] and examined which of the genes from this TRN (genes encoding TFs and target genes of TFs) are present in each of 30 fully sequenced bacteria (supplementary Table 1 in Additional data file 1). All these bacteria belong to the γ-proteobacteria, as does *E. coli *K12. By focusing on such a short evolutionary timescale, we gain insight into the dynamics of the evolution of the TRN, which is different from the insight that can be reached by looking at more distantly related organisms [10]. The bacteria we examined can be further divided into two equally sized groups based on their evolutionary distance from *E. coli *K12: the first group contains organisms that, like *E. coli *K12, belong to the Enterobacteriaceae family; and the second group contains bacteria that belong to the same class as *E. coli *K12 (γ-proteobacteria), but are more distant relatives of *E. coli *K12 and do not belong to the Enterobacteriaceae family. We divided the TFs from the TRN of *E. coli *K12 into three groups based on their presence in the other organisms (see Materials and methods): the first group included those TFs that are present in all the examined organisms ('widely present'); the second group included those TFs that are present in all Enterobacteriaceae, but are absent from some of the more distantly related non-Enterobacteriaceae ('entero-present'); and the third group included those TFs that are already absent in some of the more closely related Enterobacteriaceae genomes ('entero-absent').

### Repressors with many targets are more conserved than activators with many targets

Only 13 of the 143 TFs examined (9.1%) were found to be 'widely present', similar to the fraction of 'widely present' genes in the genome *of E. coli *K12, which is 11.5%. Fitting with the conjecture that TFs that affect more cellular functions should be more conserved, we find that out of the 13 TFs that are 'widely present', nine were previously classified in *E. coli *K12 as global regulators of transcription, or as regulators that are located at the top of the TRN hierarchy and, therefore, affect several different biological processes [9,11]. In *E. coli *K12 the 13 'widely present' TFs have, on average, a significantly higher number of targets than the 'entero-present' TFs. These, in turn, have, on average, a higher number of targets than the 'entero-absent' TFs (p ≤ 0.03 for both comparisons by one-tailed Mann-Whitney tests; Table 1). Thus, it seems that the more targets a TF has, the wider is the range of organisms in which it is conserved. However, when dividing the regulatory interactions based on mode of regulation into positive and negative, a remarkable result is found: while 'entero-present' TFs repress, on average, a significantly higher number of targets than the 'entero-absent' TFs (p ≤ 1.7 × 10^-4^), the number of targets they activate is not significantly higher than the number of targets activated by the 'entero-absent' TFs (p ≤ 0.35; Table 1).

To further investigate this phenomenon, we looked separately at TFs with a small number of targets (≤5 targets) and TFs with a large number of targets (>5 targets) (Table 2). We show that for TFs that regulate a small number of targets there is no significant difference in the presence range of activators, repressors and dual regulators; regardless of the mode of regulation, about half of these TFs are 'entero-present', while the remaining half are 'entero-absent'. Only two of the TFs that regulate a small number of targets are 'widely present'. This picture changes when examining TFs that regulate more than five targets. Even though the number of repressors and activators that regulate over five targets is rather small, a difference can be observed in their presence range (Table 2). Both repressors and activators are rarely 'widely present'. However, whereas the repressors are maintained in closely related bacteria and only 32% of them are 'entero-absent', 72% of the activators are 'entero-absent' (absent from at least two of the Enterobacteriaceae). This difference in the distribution of activators and repressors between the 'entero-present' and 'entero-absent' groups is statistically significant (p ≤ 6 × 10^-3^, by a χ^2 ^test). The dual regulators behave similarly to the repressors. However, as many of the global regulators belong to this group, members of this group are more often 'widely present'.

Why are repressors that regulate many targets less likely than activators with many targets to be absent from close relatives of *E. coli *K12? This may be due to the different outcomes of losing a repressor or an activator. In eukaryotes the transcriptional ground state is restrictive [12], due to the influence of chromatin structure on the transcription of genes. Hence, in eukaryotes most genes will not be expressed in the absence of an activator TF. In contrast, in prokaryotes the transcriptional ground state is non-restrictive and genes will normally be transcribed unless they are repressed [12]. It was argued that most of the promoters that are regulated by activators are intrinsically relatively weak [12]. Thus, the loss of an activator will often result in a partial or total loss of function of its target genes. In cases in which this is detrimental to fitness, the bacteria that lost the TF would be removed from the population by selection. However, in other cases the loss of an activator may enhance fitness; if a pathway is no longer needed, losing the TF that activates that pathway may instantaneously shut down the pathway while conserving the energy that would have otherwise been spent on transcribing the genes responsible for that pathway. On the other hand, because of the non-restrictive transcriptional ground state, the loss of a repressor might lead to constitutive expression of its target genes, resulting almost always in a reduction in fitness. This conjecture implies that the loss of a repressor must be preceded by the loss of its targets or their rewiring, while this is less crucial when losing an activator. Thus, we next turned to examine the relationship between the status of a TF (absent/present) and the status of its targets.

### Repressors, more than activators, are rarely lost while their targets remain in the genome

**Figure 2:**
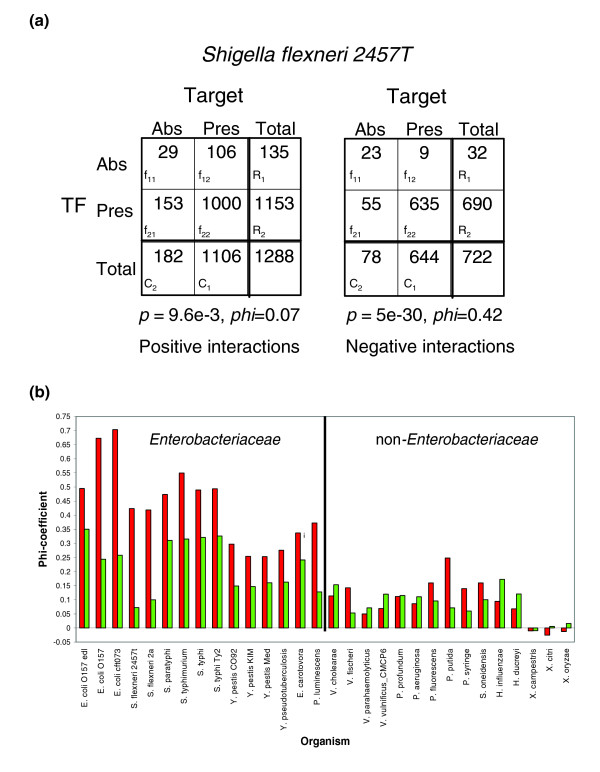
Association between the status of TFs and targets. **(a) **Contingency tables of the presence or absence of TFs and their targets in *S. flexneri *2457T for both positive and negative regulatory interactions. The significance of the associations was calculated using the χ^2 ^test. The association is stronger for negative regulatory interactions than it is for positive regulatory interactions. In a far larger fraction of positive than negative regulatory interactions, the TF is absent while the targets remain in the genome. **(b) **The strength of association between the presence or absence of TFs and that of their targets, as determined by the *phi*-coefficient. The association is stronger in bacteria closer to *E. coli *K12 than in more remote bacteria for both positive and negative regulatory interactions. In closely related bacteria, negative regulatory interactions (*phi*-coefficients represented by red bars) show stronger association than positive regulatory interactions (*phi*-coefficients represented by green bars). The values contained in the 60 contingency tables for all organisms in our study and their corresponding p values and *phi*-coefficients are listed in supplementary Table 2 in Additional data file 1.

We looked at all of the regulatory interactions in *E. coli *K12, and divided them, based on mode of regulation, into 1,288 positive and 722 negative regulatory interactions. For each mode of regulation in each of the 30 organisms, we created a contingency table of size 2 × 2 that includes the counts of regulatory interactions classified by the status of both TFs and targets (absent/present) (see Materials and methods; Figure 2a). Using the χ^2 ^test we evaluated for each of the contingency tables whether the association between the status of the targets and the status of the TFs is statistically significant. We also calculated the strength of this association by calculating the *phi*-coefficient (see Materials and methods). The values contained in all 60 contingency tables and their corresponding χ^2 ^p values and *phi*-coefficients are listed in the supplementary Table 2 in Additional data file 1. In the Enterobacteriaceae, which are more closely related to *E. coli *K12, we find for both positive and negative regulatory interactions that there is always a statistically significant association between the status of the TFs and the status of their targets (p values of the χ^2 ^tests range between 1.2e-79 and 9.6e-3). In all cases, the probability that a TF is absent when its targets are still present is lower than its probability to be absent when its targets are also absent. Yet, it is striking that in all of the 15 Enterobacteriaceae the *phi*-coefficient is higher for negative interactions than it is for positive interactions (Figure 2b). Thus, the association between the presence or absence of the TFs and their targets is weaker for positive regulatory interactions than it is for negative regulatory interactions. One reason for the differences found in the strength of association is that, in the Enterobacteriaceae, the probability for a TF to be absent while its target is maintained in the genome is higher for positive regulatory interactions than it is for negative regulatory interactions (supplementary Figure 1a in Additional data file 1). This is especially remarkable in the two *Shigella flexneri *strains. In the 2457T strain of *S. felxnari *(Figure 2a), the probability of a TF to be absent given that its target is present is 0.1 for positive regulatory interactions and only 0.01 for negative interactions. On the other hand, the probability of a target to be present given that its TF is absent is 0.79 for positive regulatory interactions and only 0.28 for negative interactions. Thus, positively regulating TFs are more likely than negatively regulating TFs to be lost from a genome, while their targets are maintained. This supports our conjecture that negatively regulated targets, but not positively regulated targets, need to be removed prior to the removal of their regulating TF.

An additional factor that affects the association between the status of TFs and that of their targets is the probability of a target to be absent while its TF is present in the genome. This probability is higher for positive regulatory interactions than it is for negative regulatory interactions (supplementary Figure 2a in Additional data file 1). We found that this trend, which is observed in both the Enterobacteriaceae and non-Enterobacteriaceae, is caused to a large extent by regulatory interactions that involve global regulators. Global regulators tend to be well conserved and regulate a large number of targets. In addition, they regulate several different biological processes. If a certain function that is regulated by a global regulator is no longer needed, the genes encoding that function may be lost. However, the global regulator may still be needed, as it regulates additional functions. Therefore, we expect to see many cases in which a global regulator is conserved while its target is absent. There are more positive than negative regulatory interactions involving global regulators in our dataset (720 and 318 interactions, respectively), which may account for the enhanced probability of an activated target to be absent while its TF remains in the genome. Once the regulatory interactions involving the 15 known global regulators of *E. coli *are removed from our analysis this enhanced probability is no longer consistent (supplementary Figure 2b in Additional data file 1). At the same time the probability of activators to be absent while their targets are present in the genome remains consistently higher than that of repressors and this trend is even enhanced (supplementary Figure 1b in Additional data file 1).

In the non-Enterobacteriaceae genomes, which are more distantly related to *E. coli *K12, we find that the association observed between the absence or presence of the TFs and that of their targets is weaker than that observed in the more closely related organisms. A significant association was found for only 11 of the 15 non-Enterobacteriaceae when considering either positive or negative regulatory interactions. In the cases in which a statistically significant association was found, the p values for the association were generally higher than those found in the Enterobacteriaceae (p values range between 2.6e-11 and 0.031), while the *phi*-coefficients were generally lower (Figure 2b; supplementary Table 2 in Additional data file 1). This indicates that, in these organisms, the association between the status of the targets and the status of the TFs is less strong. In addition, in some of the organisms that are more distantly related to *E. coli *K12, the probability of an activator to be absent from the genome while its target is present is no longer higher than that of a repressor (supplementary Figure 1 in Additional data file 1). This may be explained by the fact that the evolution of the TRN is achieved not only through changes in the repertoire of TFs and targets, but also through the rewiring of the interactions between TFs and targets (Figure 1). With the passing of time both types of changes accumulate in the TRN. It is likely, therefore, that in the distantly related organisms more targets have alternative regulation. These targets are not regulated by the same TF that regulates them in *E. coli *K12, and, therefore, their absence or presence should not affect the likelihood that that TF will be absent. Thus, the weak associations we find between the status of the TFs and targets in the non-Enterobacteriaceae, compared to *Enterobacteriaceae*, suggest that the TRNs of *E. coli *K12 and these organisms are, to a large extent, wired differently.

### Shutting down a pathway by loss of an activator

**Figure 3:**
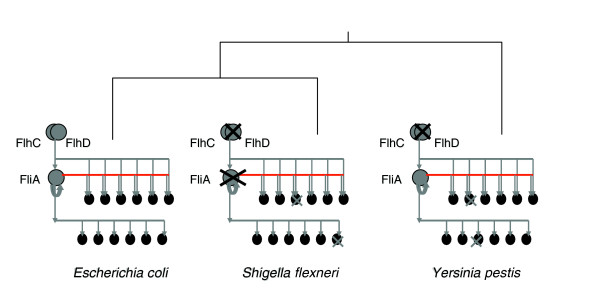
Schematic representation of the flagella biosynthesis regulon. In *E. coli *K12 the master regulator FlhDC activates the transcription of seven operons, one of which encodes the secondary activator FliA. FliA in turn activates the operons that are regulated by FlhDC, as well as additional operons. Efficient shutdown of flagella synthesis in the non-motile bacteria *S. flexneri *and *Y. pestis *is achieved by the loss of the major activator FlhDC. Nonsense mutations in genes of the regulated operons are then gradually accumulated.

We have shown in close relatives of *E. coli *K12 that activators are more likely than repressors to be lost while their targets remain in the genome. In fact, the loss of an activator may serve as an efficient means for shutting down an unnecessary pathway. As an example of this we discuss the shutdown of the flagella pathway in non-motile Enterobacteriaceae. The motility of bacteria such as *E. coli *and some of its relatives is mediated by peritrichous flagella [13]. The flagellar genes are expressed in a well controlled hierarchy, at the apex of which stands the master regulator FlhDC, a complex of two proteins, FlhC and FlhD. The FlhDC complex directly activates the transcription of seven operons, containing 34 genes. One of the genes activated by FlhDC is *fliA*, encoding the activator FliA that in turn activates additional flagellar genes (Figure 3). This pathway is conserved in all Enterobacteriaceae that grow flagella (supplementary Table 1 in Additional data file 1). The crucial role of FlhDC as a major regulator of the flagellar biosynthesis pathway was substantiated experimentally, as it has been shown that *flhD *knockout mutants are incapable of growing flagella [14]. Interestingly, in both strains of *S. flexneri *and in the three strains of *Yersinia pestis*, all of which do not grow flagella and are not motile, the FlhDC regulator is not active due to the loss of subunit FlhD, caused by a mutation in the gene encoding it (Figure 3). The *S. flexneri *strains as well as the *Y. pestis *strains have very close relatives that do grow flagella and for which FlhDC remains intact. The natural knockout mutations in *flhD *are different in the two *S. flexneri *strains from those in the three *Yersinia *strains, indicating the occurrence of two separate mutation events. in the case of *Y. pestis *an insertion of a single base has occurred, relative to the closely related *Yersinia pseudotuberculosis *sequence. This insertion resulted in a premature stop-codon being introduced into the sequence. In the two *S. flexneri *strains, the loss of *flhD *was caused by an insertion element, which deleted the first 133 bases of the gene. In a recent analysis Tominaga *et al*. [14] sequenced the *flhDC *locus of 46 non-motile *Shigella *strains. They showed that most of these strains carry non-functional copies of their *flhDC *genes, and that different strains show different mutations. In the two *S. flexneri *strains we examined, in addition to the mutation that caused the loss of FlhDC, there has also occurred a mutation causing the loss of the secondary activator FliA. Strikingly, in both *S. flexneri *and *Y. pestis*, most of the flagellar genes, which in *E. coli *K12 are regulated by FlhDC, remained intact. This, together with the observation that the *flhDC *locus has repeatedly undergone natural knockout mutations in several non-motile Enterobacteriaceae, highlights the high efficiency that is achieved by shutting down the pathway at the level of the major regulator, saving the need to knockout each target gene separately. Still, nonsense mutations in the structural genes accumulate gradually. In *S. flexneri *strain 301, seven out of the 34 genes known to be regulated by the FlhDC complex in *E. coli *underwent nonsense mutations, and their proteins are absent from the translated proteome. The same seven proteins, as well as three additional proteins, are missing from the translated proteome of the 2457T strain of *S. flexneri*. In the three *Y. pestis *strains only two to three of the flagellar proteins regulated by the FlhDC complex in *E. coli *are missing from the translated proteome. Interestingly, other than *flhD*, no common flagellar genes are missing from both *Y. pestis *and *S. flexneri*.

It is very interesting to note that all of the *S. flexneri *flagellar genes that underwent nonsense mutations are still maintained in the genome. This includes both the *flhD *gene and the *fliA *gene. Other than *flhD*, which has been truncated in *S. flexneri *and is only conserved along approximately 60% of its DNA sequence, all the flagellar genes with nonsense mutations have more than 90% sequence identity at the DNA level with their *E. coli *K12 counterparts. While *S. flexneri *is described in the Bergey's manual of systematic bacteriology [15] as a non-motile non-flagellated bacterium, Giron *et al*. [16] have identified surface appendages resembling flagella in *Shigella*. They termed these appendages flash (flagella of *Shigella*). Unlike the flagella of *E. coli *and *Salmonella *that emanate peritrichously with an average number of eight, flagellated *Shigella *produced only one polar flagellum. In addition, only 1 in 300 to 1,000 *Shigella *organisms grew flash, a frequency that is much lower than that observed in *E. coli *and *Salmonella *[16]. In the study of Giron *et al*., which was conducted before the genome sequence of *Shigella *became available, they suggested that their findings may imply that *Shigella *does grow flagella and is motile, but the regulation of the biosynthesis is different. Our findings suggest a different explanation for the observation that *Shigella *can grow flagella at low frequencies: it may be possible that the flagellar genes that are maintained in the *Shigella *genome along with the genes encoding the regulator allow a small fraction of the organisms to revert to a partially flagellated phenotype.

An additional example of the way in which loss of an activator can lead to the shutting down of an entire pathway is the loss of the maltose utilization pathway in *S. flexneri*. In *E. coli *K12 and its maltose utilizing relatives, the activator MalT induces the transcription of 10 genes of the maltose utilization pathway. This activator is absent from *S. flexneri*. It has been shown that *S. flexneri *cannot utilize maltose and that *malE*, which is one of the genes regulated by MalT in *E. coli *K12, is not expressed in *S. flexneri *[17,18]. However, the *malE *gene and the other nine maltose utilization genes are intact in the *S. flexneri *genome. These observations together show that, similar to the flagellar biosynthesis example, the shutting down of the maltose utilization pathway was achieved through the loss of the activator regulating the pathway.

## Conclusion

In this study we focused on the evolution of the TRN in a relatively large number of closely related bacteria representing a short evolutionary timescale. The TRN evolves both by removing and adding nodes (TFs and/or gene targets) and by rewiring the connections between the nodes. As evolutionary distance increases, so does the number of changes observed between two TRNs: the TRNs of two more distantly related bacteria would thus show more differences, both in the repertoire of their TFs and in the ways in which the TFs and targets are connected. We show an interesting difference in the way in which the repertoires of repressors and activators evolve. In order for a repressor to be removed from the TRN, its targets need to either acquire alternative regulation through the rewiring of the network, or be removed themselves. For this reason, among closely related bacteria we rarely observe the removal of repressors, especially those that regulate many targets, and when such changes do occur they are frequently preceded by the removal of the target genes. In contrast, we observe changes in the repertoire of activators even among TRNs of very closely related bacteria. Activators may be lost as a way of turning off a pathway. In these cases the activator may be lost prior to the loss of its targets.

## Materials and methods

### The TRN of *E. coli *K12

Data on *E. coli *K12 transcription factors and their target genes were extracted from Ma *et al *[9]. This data set includes regulatory interactions of TFs in *E. coli *K12, including the sigma factors RpoS, RpoN, RpoE and RpoH. The sigma factors were not included in the analysis because they function as part of the RNA polymerase holoenzyme [3,4], and are not considered as TFs. Interactions involving RyhB, glnL, Hfq or UidA as the regulators were also excluded because these molecules are not TFs [19-22]. In addition, all auto-regulatory interactions and all regulatory interactions for which the mode of regulation (positive, negative or dual) is unknown were also excluded. The resulting data set contains 2,285 regulatory interactions between 143 TFs and 1,048 target genes (Additional data file 2).

Of the 143 TFs included in our analysis, 15 have previously been characterized as global regulators, or as regulators that are located at the top layers of the hierarchical structure of the TRN [9,11]. Such TFs are expected to affect several biological processes and integrate between them. These TFs are: CRP, IhfA, IhfB, FNR, Hns, ArcA, FIS, LRP, PhoB, ArgP, CspA, CspE, CytR, SoxR, and DnaA.

The regulatory interactions that were collected by Ma *et al*. [9] have since been included in the RegulonDB [23] and Ecocyc [24] databases. These regulatory interactions and their mode of regulation were gathered from publications and were determined by small-scale experiments.

### Determining the presence or absence of genes from *E. coli *K12 in other γ-proteobacteria

Gene sequences were extracted from version NC_000913.1 of the *E. coli *K12 genome, and annotations of the genes were extracted from the Ecogene database [25]. The genomic and protein sequences and the annotations of the 30 genomes in supplementary Table 1 in Additional data file 1 were downloaded from the NCBI ftp server [26]. These 30 organisms can be divided into two groups, each containing 15 bacteria. The first group includes bacteria that, like *E. coli *K12, belong to the Enterobacteriaceae family. The second group contains bacteria that are not members of the Enterobacteriaceae family, but are included in the same class as *E. coli *(γ*-*proteobacteria). All amino acid sequences of the proteins encoded in *E. coli *K12 were compared to the sequences of the annotated proteins of each of the 30 organisms, using a locally installed version of the FASTA program [27]. For each protein we recorded its best hit in each of the 30 organisms and the percentage identity across the entire *E. coli *K12 protein sequence. At the DNA level, each *E. coli *K12 protein-coding gene was compared to the complete genomic sequence of each of the 30 organisms, and the best hit and percentage identity were recorded for each organism.

For each gene in *E. coli *K12 and each organism, we compared the genomic location of the gene encoding the best hit at the protein level to the genomic location of the best hit at the DNA level. If in a certain genome the best hit at the protein level is located in the same location as the best hit at the DNA level, we consider the *E. coli *K12 gene and protein to be present in that genome. If the location of the protein best hit is different from that of the DNA best hit, we regard this protein as present in the genome if the percentage identity at the protein level is at least 40%.

We expect that for the proteins that are present in the different genomes the average percent identity will decrease as the evolutionary distance from *E. coli *K12 increases. The percentage of *E. coli *K12 genes that are maintained in a genome can be used as a measure of the distance of that genome from *E. coli *K12. Thus, if our threshold is reasonable, we expect to find a strong correlation between the average percent identity and the percentage of the *E. coli *K12 proteins that we annotated as present in the different organisms. Indeed, the Pearson correlation coefficient between the percentage of proteins that, according to our threshold, are present in the genome and their average percent identity is 0.97 (supplementary Table 1 in Additional data file 1). In contrast, the average percent identity of the best hits for the proteins that did not pass our threshold does not change with the evolutionary distance from *E. coli *K12 (Pearson correlation of -0.05; supplementary Table 1 in Additional data file 1). We therefore conclude that our threshold allows the separation of those proteins that are present in a genome from hits that are generated by chance.

Our method is different from the best bidirectional hit method that is commonly used to assign orthologs across large evolutionary time scales. We believe that when comparing closely related organisms for assigning a status of absence or presence to a gene our method is more suitable. However, to make sure that our results were not strongly affected by our assignment methodology we compared it to the best bidirectional hit method. We found that when comparing all of the proteins of *E. coli *K12 across the 30 organisms examined, the methods assign the genes differently in less than 4% of the cases.

### Classifying TFs based on their presence in the various organisms

**Figure 4:**
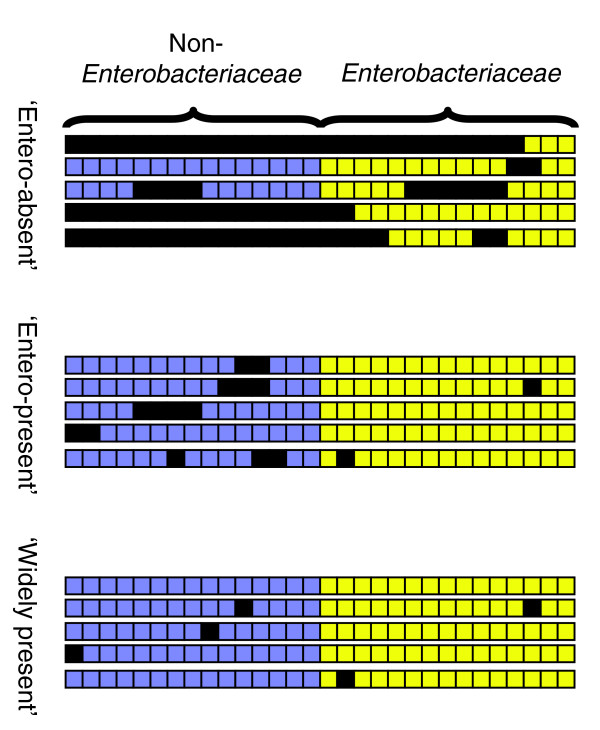
Classifying *E. coli *K12 transcription factors into three groups based on their conservation across *E. coli *K12 close and remote relatives. The first group of TFs includes TFs that appear in most of the 30 bacteria in our study ('widely present'). A TF was included in this group if it appears in at least 14 of the 15 Enterobacteriaceae and in at least 14 of the 15 non-Enterobacteriaceae genomes. The second group includes those TFs that are present in all closely related Enterobacteriaceae genomes and are absent only from the more distantly related non-Enterobacteriaceae organisms ('entero-present'). A TF was classified into this group if it was present in at least 14 of the 15 Enterobacteriaceae and was absent from two or more of the 15 non-Enterobacteriaceae. The last group includes those TFs that are absent from some of the most closely related Enterobacteriaceae. TFs were classified into this group if they are absent from at least two of the 15 Enterobacteriaceae ('entero-absent'). For each of the three groups, five examples of conservation patterns of TFs that would be classified into that group are illustrated. Yellow and purple boxes represent presence of a TF in Enterobacteriaceae and non-Enterobacteriaceae, respectively. Black boxes indicate absence of the TF from an organism. Each column illustrates an example of presence/absence pattern that would result in classification of a TF in one of the three classes.

The TFs of *E. coli *K12 were classified into three groups based on their presence across the various organisms. The classification criteria and the description of the three groups are detailed in Figure 4. The procedure used aimed to minimize misclassifications due to sequencing errors; for example, the first group of TFs includes those that are present in most organisms (termed 'widely present'). To limit the effects of sequencing errors in individual genomes, we did not require the TF to be present in all organisms in order to be classified into this group, but required it to appear in at least 14 of the 15 Enterobacteriaceae and in at least 14 of the 15 non-Enterobacteriaceae genomes. The classification of the 143 TFs into the three groups can be found in Additional data file 3.

### Evaluating the association between the status (present/absent) of the TFs and their targets

Regulatory interactions from *E. coli *K12 were divided based on their mode of regulation into positive and negative interactions. For each mode of regulation in each of the 30 organisms a contingency table of size 2 × 2 was created. Each contingency table contains the number of regulatory interactions in each of the four following categories: both the TF and its target are present in the genome (TF_pres_, targ_pres_); the TF is absent but its target is present (TF_abs_, targ_pres_); the TF is present but its target is absent (TF_pres_, targ_abs_); and both the TF and its target are absent (TF_abs_, targ_abs_). For each contingency table we carried out a χ^2 ^test, testing the null hypothesis that the status of the targets (absent/present) and the status of the TFs are not associated. Rejection of the null hypothesis with p ≤ 0.05 implied a statistically significant association. We also estimated the strength of association by the *phi*-coefficient. The *phi*-coefficient is a derivative of the χ^2 ^test. It is calculated as:

Phi=f11⋅f22−f12⋅f21C1C2R1R2
 MathType@MTEF@5@5@+=feaafiart1ev1aaatCvAUfeBSjuyZL2yd9gzLbvyNv2Caerbhv2BYDwAHbqedmvETj2BSbqee0evGueE0jxyaibaiKI8=vI8tuQ8FMI8Gi=hEeeu0xXdbba9frFj0=OqFfea0dXdd9vqai=hGuQ8kuc9pgc9s8qqaq=dirpe0xb9q8qiLsFr0=vr0=vr0dc8meaabaqaciGacaGaaeqabaqadeqadaaakeaacaWGqbGaamiAaiaadMgacqGH9aqpdaWcaaqaaiaadAgadaWgaaWcbaGaaGymaiaaigdaaeqaaOGaeyyXICTaamOzamaaBaaaleaacaaIYaGaaGOmaaqabaGccqGHsislcaWGMbWaaSbaaSqaaiaaigdacaaIYaaabeaakiabgwSixlaadAgadaWgaaWcbaGaaGOmaiaaigdaaeqaaaGcbaWaaOaaaeaacaWGdbWaaSbaaSqaaiaaigdaaeqaaOGaam4qamaaBaaaleaacaaIYaaabeaakiaadkfadaWgaaWcbaGaaGymaaqabaGccaWGsbWaaSbaaSqaaiaaikdaaeqaaaqabaaaaaaa@4DDE@

where *f11*, *f12*, *f21*, and *f22 *represent the counts appearing in the four cells of the 2 × 2 contingency tables, *C1 *and *C2 *represent the column sums of the values and *R1 *and *R2 *represent their row sums (Figure 2a).

*Phi *values can range from -1 to 1. The further the value is from zero, the stronger the association. Positive values indicate a positive association, while negative values indicate an inverse association. Thus, in our case a value of 1 would mean that there is complete agreement between the status of the TF and that of its targets. In such a case if the TF is present, all its targets would be present, and if a TF is absent, all its targets would be absent. A value of -1 would indicate a negative association. All the targets of an absent TF would be present and *vice versa*.

Our method of assigning orthologous relations depends on analyzing conservation at both the protein and the DNA levels. For this reason the 95 regulatory interactions in which the target is an RNA gene (tRNA, rRNA or ncRNA) were not considered in this analysis. These 95 interactions are marked by an asterisk in Additional data file 2.

## Additional data files

The following additional data are available with the online version of this paper. Additional data file 1 contains supplementary figures and tables: supplementary Table 1 lists information regarding the 30 organisms used in the study; supplementary Table 2 lists the association between the status of TFs and the status of their targets; supplementary Figure 1 shows the probability of activators and repressors to be absent in the different genomes, while their targets are present; supplementary Figure 2 shows the probability of repressed and activated targets to be absent from the different genomes, while their regulating TFs are present. Additional data file 2 lists the regulatory interactions included in this study. Additional data file 3 lists the classification of TFs into three groups based on their presence in the different organisms.

## Supplementary Material

Additional data file 1 Supplemetary Table 1 lists information regarding the 30 organisms used in the study. Supplementary Table 2 lists the association between the status of TFs and the status of their targets. Supplementary Figure 1 shows the probability of activators and repressors to be absent in the different genomes, while their targets are present. Supplementary Figure 2 shows the probability of repressed and activated targets to be absent from the different genomes, while their regulating TFs are present. Click here for file

Additional data file 2 Regulatory interactions included in this study. Click here for file

Additional data file 3 Classification of TFs into three groups based on their presence in the different organisms. Click here for file

## Figures and Tables

**Table 1:** Average number of targets of transcription factors classified based on conservation range

Type of targets	Entero-absent TFs	Entero-present TFs	Widely present TFs*
All targets^†^	6.7 ± 8.9	13.9 ± 23.5	66.6 ± 85.2
Repressed targets	1.4 ± 2.6	6.2 ± 11.5	16.6 ± 17.8
Activated targets	5.1 ± 9	6.7 ± 12.8	42.3 ± 62.2

*The large standard deviations are due to several global TFs that regulate hundreds of targets. ^†^Total targets, including repressed targets, activated targets anddually regulated targets.

**Table 2:** Presence of *E. coli *K12 transcription factors in close and remote relatives

TF type	In *E. coli *K12	Entero-absent*	Entero- present^†^	Widely present^‡^
All TFs	143	65 (45.5%)	65 (45.5%)	13 (9%)
				
		TFs that regulate ≤ 5 targets		
All	71	34 (48%)	35 (49%)	2 (3%)
Activators	39	22 (56%)	16 (41%)	1 (3%)
Repressors	27	11 (41%)	15 (56%)	1 (3%)
Dual regulators	5	1 (20%)	4 (80%)	0 (0%)
				
		TFs that regulate >5 targets		
All	72	31 (43%)	30 (42%)	11 (15%)
Activators^§^	29	21 (72%)	7 (24%)	1 (4%)
Repressors^¶^	22	7 (32%)	13 (59%)	2 (9%)
Dual regulators^¥^	21	3 (14%)	10 (48%)	8 (38%)

*Absent from *Enterobacteriaceae*. ^†^Present in *Enterobacteriaceae *but absent from other γ-proteobacteria. ^‡^Present in most γ-proteobacteria. ^§^TFs are included in this group if they activate more than five targets. If the same TF also represses targets (dual regulator), it is included in this group only if the number of targets it activates is more than twice the number of repressed targets, and if the number of repressed targets is not larger than five. ^¶^TFs are included in this group if they repress more than five targets. If the TF is a dual regulator, it is included in this group only if the number of targets it represses is more than twice the number of activated targets, and if the number of activated targets is not larger than five. ^¥^TFs are included in this group if they regulate more than five genes but cannot be assigned to the previous two groups.
