# Supplementary material for: Co-evolution of transcription factors and their targets depends on mode of regulation
Source: Genome Biol. 2006 Jul 19;7(7):R62. doi: 10.1186/gb-2006-7-7-r62 (PMC1779565; doi:10.1186/gb-2006-7-7-r62)
Supplement: Additional data file 1 — Supplemetary Table 1 lists information regarding the 30 organisms used in the study. Supplementary Table 2 lists the association between the status of TFs and the status of their targets. Supplementary Figure 1 shows the probability of activators and repressors to be absent in the different genomes, while their targets are present. Supplementary Figure 2 shows the probability of repressed and activated targets to be absent from the different genomes, while their regulating TFs are present. [file gb-2006-7-7-r62-S1.pdf]

**Supplementary Table I.** Organisms used in the study

| Organism | # of protein-coding genes | # (%) of *E. coli* K12 protein-coding genes present in organism | Average percent identity of Present *E. coli* K12 proteins[a] | Average percent identity of Absent E. coli K12 proteins[b] | Pathogenicity | Motility |
|---|---|---|---|---|---|---|
| *Escherichia coli* O157:H7 EDL933 | 5324 | 3849 (92%) | 97 | 20 | Hamburger disease | Yes |
| *Escherichia coli* O157:H7 | 5341 | 3835 (92%) | 96 | 20 | Hamburger disease | Yes |
| *Escherichia coli* CFT073 | 5379 | 3689 (88%) | 96 | 19 | Urinary tract infections | Yes |
| *Shigella flexneri* 2a strain 2457T | 4068 | 3304 (79%) | 96 | 18 | Shigellosis | No |
| *Shigella flexneri* 2a strain 301 | 4441 | 3355 (80%) | 96 | 18 | Shigellosis | No |
| *Salmonella paratyphi* | 4092 | 3127 (75%) | 86 | 19 | Paratyphoid fever | Yes |
| *Salmonella typhimirum* LT2 | 4527 | 3246 (78%) | 86 | 20 | Gastroenteritis / Typhoid like disease in mice | Yes |
| *Salmonella typhi* strain CT18 | 4758 | 3162 (76%) | 85 | 20 | Typhoid fever | Yes |
| *Salmonella typhi* strain Ty2 | 4318 | 3140 (75%) | 86 | 20 | Typhoid fever | Yes |

| | | | | | | |
|---|---|---|---|---|---|---|
| *Yersinia pestis* CO92 | 4067 | 2381 (57%) | 71 | 20 | Plague | No |
| *Yersinia pestis* KIM | 4086 | 2375 (57%) | 71 | 20 | Plague | No |
| *Yersinia pestis* biovar Mediaevails | 4142 | 2394 (57%) | 71 | 20 | Plague | No |
| *Yersinia pseudotuberculosis* | 4038 | 2458 (59%) | 72 | 20 | Gastroenteritis and tuberculosis like infection | Yes |
| *Erwinia carotovora* | 4472 | 2497 (60%) | 71 | 20 | Soft rot disease of potatoes | Yes |
| *Photorhabdus luminescens* | 4683 | 2042 (49%) | 69 | 19 | Toxemia and septicemia in insects | Yes |
| *Vibrio cholerae* | 3835 | 1568 (37%) | 61 | 19 | Cholerae | Yes |
| *Vibrio fischeri* ES114 | 3802 | 1540 (37%) | 59 | 20 | A symbiont in fish and squids. Is responsible for light generation | No |
| *Vibrio parahaemolyticus* | 4832 | 1671 (40%) | 60 | 20 | food poisoning (gastroenteritis) | Yes |
| *Vibrio vulnificus* CMCP6 | 4488 | 1639 (39%) | 59 | 20 | Opportunistic pathogen. Causes gastroenteritis , wound infections, and primary septicemia. | Yes |

| | | | | | | |
|---|---|---|---|---|---|---|
| *Photobacterium profundum* SS9 | | 1731 (41%) | 60 | 21 | Non-pathogenic, A tractable model system for studies of low temperature and high pressure adaptation | Yes |
| *Pseudomonas aeruginosa* | 5567 | 1475 (35%) | 55 | 21 | Opportunistic infections in immuno-compromised patients | Yes |
| *Pseudomonas fluorescens* Pf-5 | 6137 | 1427 (34%) | 55 | 22 | Nonpathogenic , inhabits soil, water and plant surface environments | Yes |
| *Pseudomonas putida* KT2440 | 5350 | 1363 (33%) | 55 | 21 | Non-pathogenic. | Yes |
| *Pseudomonas syringae* | 5608 | 1350 (32%) | 55 | 21 | Plant pathogens | Yes |
| *Shewanella oneidensis* | 4472 | 1369 (33%) | 58 | 20 | Rare opportunistic infections | Yes |
| *Haemophilus influenzae* | 1657 | 1159 (28%) | 60 | 17 | Meningitis, septicemia, otitis media, sinusitis and chronic bronchitis | No |
| *Haemophilus ducreyi* 35000HP | 1717 | 974 (23%) | 60 | 16 | Sexually-transmitted chancroid and enhances HIV transmission | No |

| | | | | | | |
|---|---|---|---|---|---|---|
| *Xanthomonas campestris* | 4181 | 978 (23%) | 52 | 20 | Black rot in plants | Yes |
| *Xanthomonas citri* | 4427 | 981 (23%) | 52 | 20 | Plant pathogen. Affects citrus plants | ? |
| *Xanthomonas oryzae* KACC10331 | 4637 | 819 (20%)l | 53 | 19 | Plant pathogen. Affects rice plants by causing leaf blight | No |

[a]Average percent identity between the ortholgous proteins found in the genome and their *E. coli* K12 counterparts.

[b]Average percent identity between the best hits that were found for in the genome for each *E. coli* K12 protein, for which no ortholog was identified

**Supplementary Table 2.** Association between the status of TFs and the status of their targets

| Organism | Positive regulatory interactions | | | | | | Negative regulatory interactions | | | | | |
|---|---|---|---|---|---|---|---|---|---|---|---|---|
| | $TF_{-},tar_{-}$[a] | $TF_{-},tar_{+}$[b] | $TF_{+},tar_{-}$[c] | $TF_{+},tar_{+}$[d] | p-value[e] | Phi[f] | $TF_{-},tar_{-}$[a] | $TF_{-},tar_{+}$[b] | $TF_{+},tar_{-}$[c] | $TF_{+},tar_{+}$[d] | p-value[e] | *Phi*[f] |
| *Escherichia coli* O157:H7 EDL933 | 17 | 5 | 79 | 1187 | 2.8e-36 | 0.35 | 12 | 11 | 12 | 687 | 2.9e-40 | 0.49 |
| *Escherichia coli* O157:H7 | 9 | 5 | 76 | 1198 | 2.3e-18 | 0.24 | 22 | 5 | 16 | 679 | 4.8e-73 | 0.67 |
| *Escherichia coli* CFT073 | 9 | 5 | 68 | 1206 | 2.2e-20 | 0.26 | 22 | 5 | 13 | 682 | 1.2e-79 | 0.7 |
| *Shigella flexneri* 2a strain 2457T | 29 | 106 | 153 | 1000 | 9.6e-3 | 0.07 | 23 | 9 | 55 | 635 | 5e-30 | 0.42 |
| *Shigella flexneri* 2a strain 301 | 34 | 101 | 157 | 996 | 3.4e-4 | 0.1 | 23 | 10 | 54 | 635 | 2.4e-29 | 0.41 |
| *Salmonella paratyphi* | 54 | 39 | 160 | 1035 | 7.2e-29 | 0.31 | 28 | 11 | 49 | 634 | 4.8e-37 | 0.47 |
| *Salmonella typhimirum* LT2 | 50 | 37 | 147 | 1054 | 1.1e-29 | 0.32 | 29 | 9 | 37 | 647 | 2.6e-49 | 0.55 |
| *Salmonella typhi* strain CT18 | 52 | 37 | 149 | 1050 | 8.6e-31 | 0.32 | 28 | 9 | 49 | 636 | 1.6e-39 | 0.49 |
| *Salmonella typhi* strain Ty2 | 53 | 36 | 151 | 1048 | 1.2e-31 | 0.33 | 28 | 9 | 48 | 637 | 4.1e-40 | 0.49 |
| *Yersinia pestis* CO92 | 137 | 109 | 386 | 656 | 8.5e-08 | 0.15 | 49 | 23 | 153 | 497 | 1.4e-15 | 0.30 |
| *Yersinia pestis* KIM | 143 | 111 | 394 | 640 | 1.4e-07 | 0.15 | 50 | 30 | 164 | 478 | 8.8e-12 | 0.25 |
| *Yersinia pestis* biovar Mediaevails | 148 | 112 | 383 | 645 | 8.7e-09 | 0.16 | 53 | 31 | 170 | 468 | 1.1e-11 | 0.25 |
| *Yersinia pseudotuberculosis* | 150 | 111 | 385 | 642 | 4.9e-09 | 0.16 | 50 | 26 | 161 | 485 | 1.3e-13 | 0.28 |

| | | | | | | | | | | | | |
|---|---|---|---|---|---|---|---|---|---|---|---|---|
| *Erwinia carotovora* | 107 | 65 | 321 | 795 | 4.4e-18 | 0.24 | 45 | 24 | 115 | 538 | 1.4e-19 | 0.34 |
| *Photorhabdus luminescens* | 163 | 85 | 515 | 525 | 4.4e-06 | 0.13 | 108 | 27 | 193 | 394 | 1.3e-23 | 0.37 |
| *Vibrio cholerae* | 338 | 109 | 507 | 334 | 3.5e-08 | 0.15 | 111 | 57 | 292 | 262 | 2.2e-3 | 0.11 |
| *Vibrio fischeri* ES114 | 277 | 130 | 551 | 330 | 0.055 | 0.05 | 87 | 37 | 307 | 291 | 1.3e-4 | 0.14 |
| *Vibrio parahaemolyticus* | 263 | 137 | 517 | 371 | 0.011 | 0.07 | 64 | 53 | 290 | 315 | 0.18 | 0.05 |
| *Vibrio vulnificus* CMCP6 | 282 | 124 | 501 | 381 | 1.5e-05 | 0.12 | 61 | 46 | 291 | 324 | 0.064 | 0.07 |
| *Photobacterium profundum SS9* | 254 | 106 | 539 | 389 | 3.6e-05 | 0.12 | 67 | 42 | 281 | 332 | 2.6e-3 | 0.11 |
| *Pseudomonas aeruginosa* | 310 | 144 | 475 | 359 | 6.9e-05 | 0.11 | 169 | 118 | 218 | 217 | 0.021 | 0.09 |
| *Pseudomonas fluorescens* Pf-5 | 385 | 199 | 398 | 306 | 5.9e-4 | 0.1 | 224 | 143 | 160 | 195 | 1.7e-05 | 0.16 |
| *Pseudomonas putida* KT2440 | 310 | 159 | 482 | 337 | 0.01 | 0.07 | 181 | 75 | 209 | 257 | 2.6e-11 | 0.25 |
| *Pseudomonas syringae* | 332 | 161 | 488 | 307 | 0.031 | 0.06 | 202 | 120 | 195 | 205 | 1.7e-4 | 0.14 |
| *Shewanella oneidensis* | 312 | 118 | 536 | 322 | 3.2e-4 | 0.1 | 168 | 83 | 237 | 234 | 1.8e-05 | 0.16 |
| *Haemophilus influenzae* | 416 | 87 | 526 | 259 | 5.6e-10 | 0.17 | 126 | 50 | 333 | 213 | 0.011 | 0.09 |
| *Haemophilus ducreyi* 35000HP | 504 | 88 | 525 | 171 | 1.5e-05 | 0.12 | 193 | 52 | 346 | 131 | 0.068 | 0.07 |
| *Xanthomonas campestris* | 499 | 147 | 501 | 141 | 0.73 | -0.01 | 347 | 126 | 185 | 64 | 0.79 | -0.01 |
| *Xanthomonas citri* | 484 | 143 | 507 | 154 | 0.83 | 0.01 | 341 | 127 | 191 | 63 | 0.5 | -0.03 |

| | | | | | | | | | | | | |
|---|---|---|---|---|---|---|---|---|---|---|---|---|
| *Xanthomonas oryzae* KACC10331 | 521 | 112 | 531 | 124 | 0.57 | 0.02 | 384 | 97 | 195 | 46 | 0.73 | -0.01 |

[a]Number of positive or negative *E. coli* K12 regulatory interactions for which both the TF and its target are absent from this genome

[b]Number of positive or negative *E. coli* K12 regulatory interactions for which the TF is absent and its target is present.

[c]Number of positive or negative *E. coli* K12 regulatory interactions for which the TF is present and its target is absent.

[d]Number of positive or negative *E. coli* K12 regulatory interactions for which both the TF and its target are present.

[e]Two-sided Pearson $\chi^2$ test (df=1) was conducted to test the null hypothesis that the status of the targets (absent / present) and the status of the TFs are not associated. The p-values of the $\chi^2$ test are listed. Rejection of the null hypothesis with $p \leq 0.01$ implied a statistical significant association.

[f]The *phi*-coefficient was calculated to measure the strength of the association between the status of the targets and the status of the TFs.

**Supplementary Figure 1 (a)**

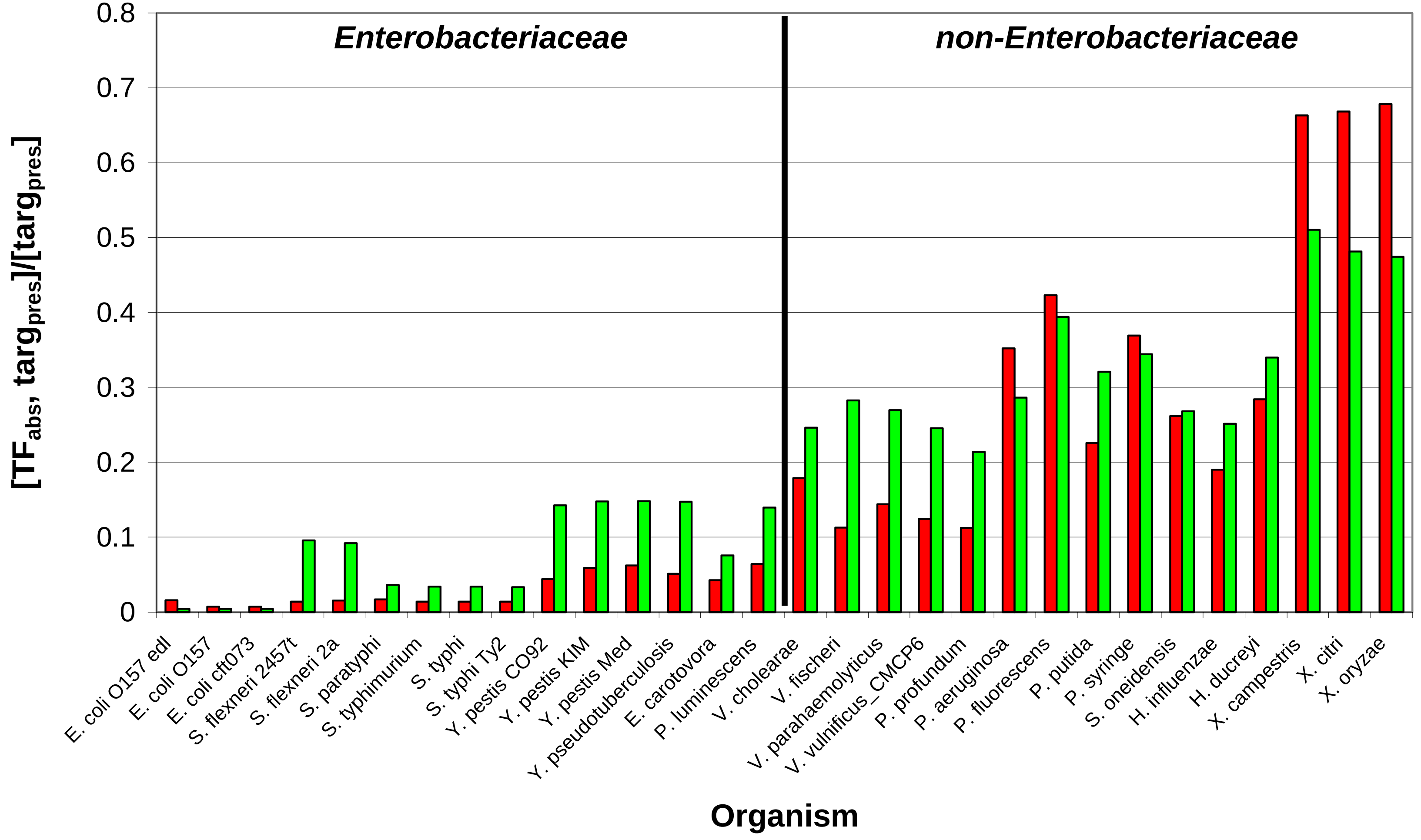

**Supplementary Figure 1 (b)**

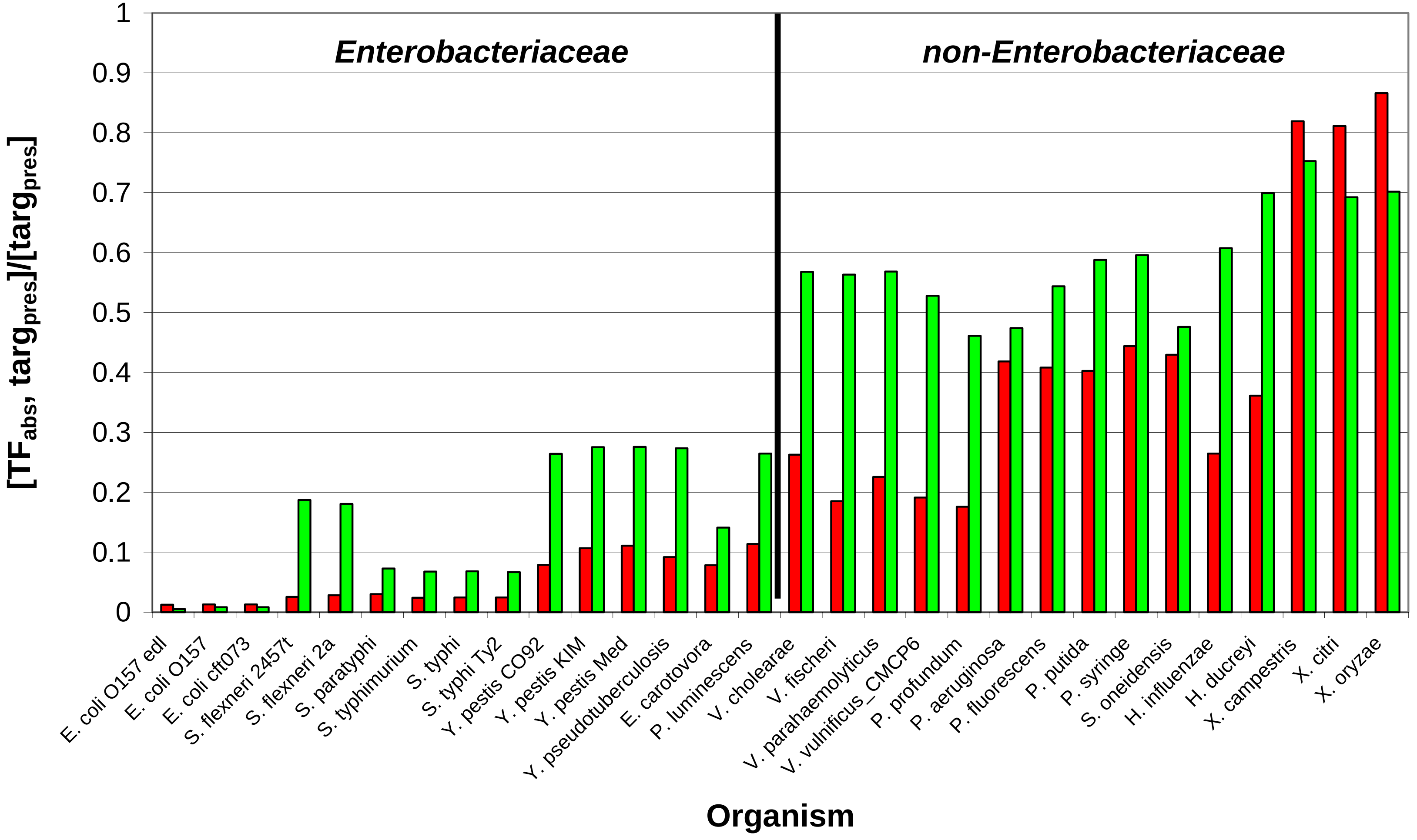

**Supplementary Figure 2 (a)**

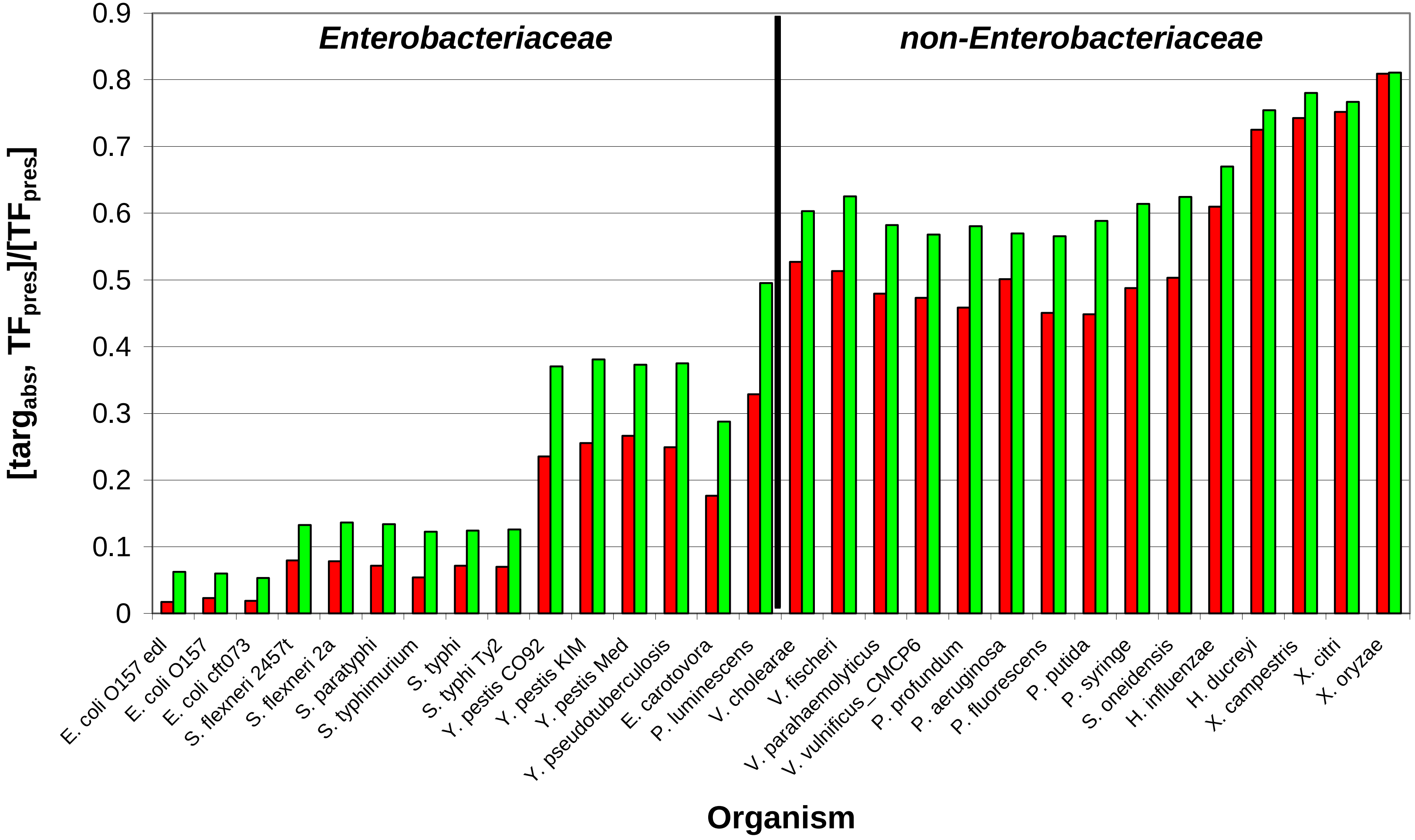

**Supplementary Figure 2 (b)**

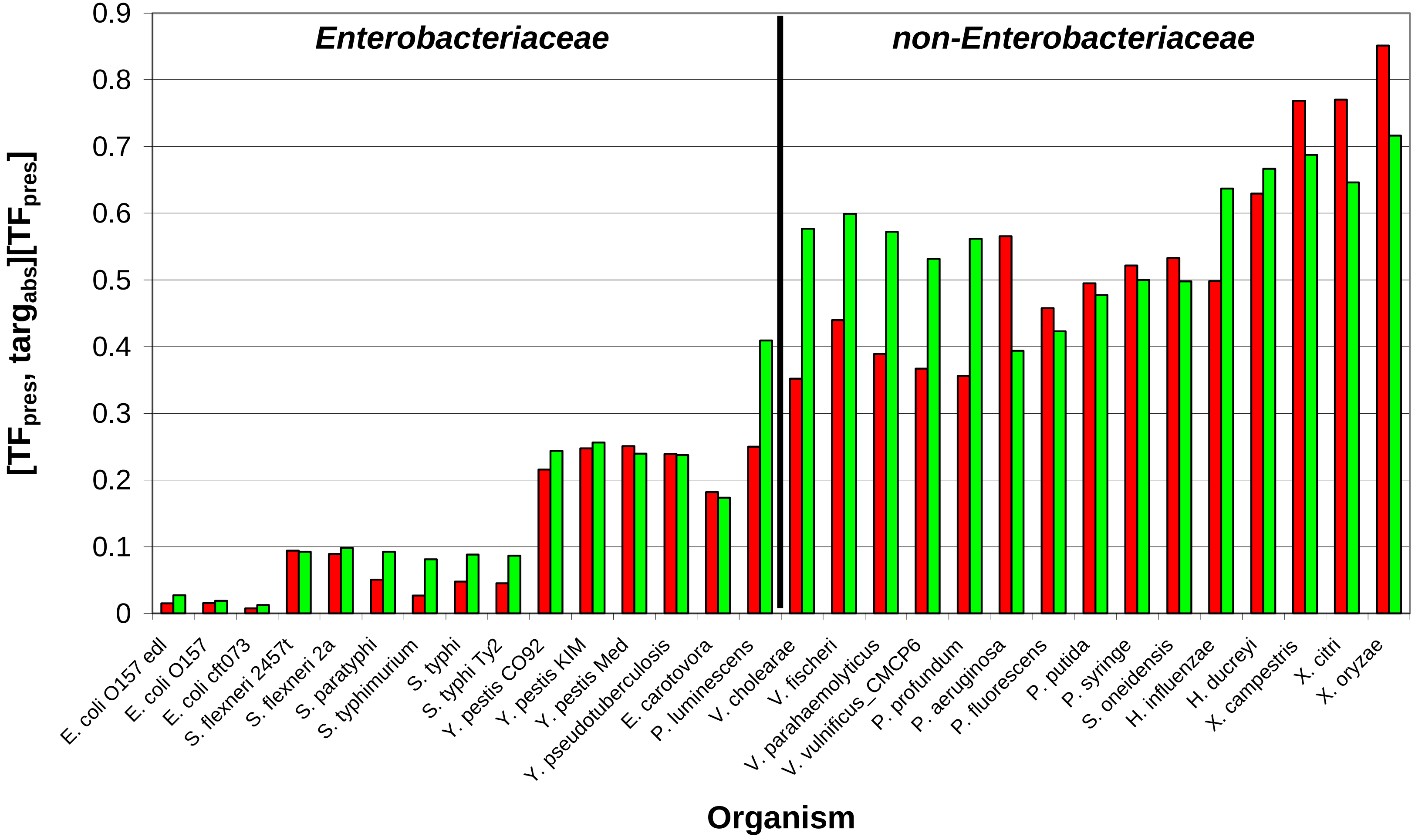

## Figure legends

**Supplementary Figure 1.** Probability of a TF to be absent given that its targets are present. (a) Calculated for all positive (green bars) and negative (red bars) regulatory interactions. (b) Calculated after regulatory interactions mediated by global regulators were removed.

**Supplementary Figure 2.** Probability of target to be absent given its TF is present. (a) Calculated for all positive (green bars) and negative (red bars) regulatory interactions. (b) Calculated after regulatory interactions mediated by global regulators were removed.
